# Cation-Selective Actuator–Sensor Response of Microcrystalline Cellulose Multi-Walled Carbon Nanotubes of Different Electrolytes Using Propylene Carbonate Solvent

**DOI:** 10.3390/polym16030339

**Published:** 2024-01-26

**Authors:** Fred Elhi, Quoc Bao Le, Rudolf Kiefer

**Affiliations:** 1 Intelligent Materials and Systems Lab, Institute of Technology, University of Tartu, Nooruse 1, 50411 Tartu, Estonia; elhi.fred@gmail.com; 2 Conducting Polymers in Composites and Applications Research Group, Faculty of Applied Sciences, Ton Duc Thang University, Ho Chi Minh City 700000, Vietnam; lequocbao@tdtu.edu.vn

**Keywords:** MC-MCNT fiber, three electrolytes, linear actuation, PC solvent, cation-selective sensor

## Abstract

Microcrystalline cellulose (MC) with 50 wt.% multi-walled carbon nanotube (MCNT) composites is obtained through extrusion, forming MC-MCNT fiber. In this study, we concentrate on three different electrolytes in propylene carbonate (PC) which have the same anions (TF^−^, trifluoro-methanesulfonate CF_3_SO_3_^−^) but different cations, EDMI^+^ (1-ethyl-2,3-dimethylimidazolium), Li^+^ (lithium ion), and TBA^+^ (tetrabutylammonium). Cyclic voltammetry and square wave potential steps, in combination with linear actuation measurements in a potential range of 0.7 V to −0.2 V, were conducted. Our goal in this work was to establish a cation-selective actuator–sensor device capable of distinguishing different cations. The linear actuation of MC-MCNT fiber had its main expansion at discharge due to the incorporation of TF^−^ in the MC-MCNT fiber with the cations. In the following order, TBA^+^ > EDMI^+^ > Li^+^ had the best stress, strain, charge density, diffusion coefficients, and long-term stability. Chronopotentiometric measurements revealed that the cations in the PC solvent can be differentiated by their ion sizes. Further characterization of the MC-MCNT fiber was completed using scanning electron microscopy (SEM), energy-dispersive X-ray spectroscopy (EDX), and FTIR and Raman spectroscopy.

## 1. Introduction

Microcrystalline cellulose, as a green alternative for a binder with electroactive materials such as multi-walled carbon nanotubes (MCNTs), is being considered as a potential move toward biodegradable composite materials. Most research is focused on obtaining cellulose carbon nanotube composites that function as actuators [1,2], supercapacitors [3,4], or sensors [5], with some having multifunctional applications [6]. Microcrystalline cellulose (MC) has several advantages in comparison to synthetic polymer binders; it is sustainable, naturally abundant, inexpensive, and able to be dissolved in Ionic liquids (ILs) [7]. With the addition of MCNT, a stable suspension is obtained. Such a cellulose IL MCNT suspension can be formed in fiber through electrospinning [8] or obtained via extrusion, forming MC-MCNT fiber in anti-solvent (water) [9], as was carried out in this work. Recent research using MC-MCNT fiber in aqueous [10] or organic electrolytes [11] revealed that the chosen potential range plays a role in which actuation direction, either expansion at negative charging or expansion at positive charging, is obtained.

The general actuation mechanism using MCNTs in MC-MCNT fibers follows a non-faradaic process [12] with an charge injection in the MCNTs, building up an electrical double layer (EDL) and changing the C–C bond length [13]. In addition to the stable actuation response, recent research [14] revealed that changes in the potential range of anions and cations can be differentiated and electrochemically sensed in aqueous electrolytes. The sensing of ions in aqueous electrolytes using cellulose MCNTs or even only MCNT electrodes has been shown [15], along with the detection of ammonia [16] or heavy metals [17]. Ion-selective electrodes have been used in various research projects with aqueous solvents [18], while the detection in an organic solvent has rarely been investigated [19].

Therefore, our first aim in this work is to use MC-MCNT fiber to investigate its linear actuation properties in propylene carbonate (organic solvent) in three different electrolytes and using the same anion (TF^−^) with three different cations: EDMI^+^ (EDMITF), Li^+^ (LiTF), and TBA^+^ (TBATF). Cyclic voltammetry and square wave potential steps at different frequencies were conducted to evaluate actuation direction and actuation properties in stress, strain, charge density, and long-term stability. Our second aim is to orientate selected cations to see if they can be differentiated in electrochemical sensors by applying chronopotentiometric measurements. Further, we want to understand which ion properties matter, specifically, the size of the ions or their hydrophilic/hydrophobic interactions, in order to determine the differences in cations and formulate a unique cation-selective electrode (MC-MCNT fiber) that will be applicable in an organic solvent in a dual actuator–sensor application.

The MC-MCNT fiber is characterized using SEM images, FTIR, Raman spectroscopy, and EDX spectroscopy.

## 2. Materials and Methods

### 2.1. Microcrystalline Cellulose MWCNT Formulation

Microcrystalline cellulose (MC) (average particle size of 20 µm, purchased from Sigma-Aldrich, Leverkusen, Germany) was dissolved with the Ionic liquid (IL) 1-ethyl-3-methylimidazolium chloride (EMIMCl, >97%). The MCNTs (MWCNT, Baytubes^®^; length > 1 µm, inner diameter 4 nm, and outer diameter 12 nm) were purchased from Bayer Material Science (BMS, Leverkusen, Germany) and used as supplied. After 12 h stirring at 85 °C, the MCNTs (MCNT, 50 wt.%) were added to the suspension and ultra-sonicated at room temperature for 30 min. The mixture was then placed in a syringe (760 µm inner diameter) and extruded in anti-solvent (Milli-Q+, Tallinn, Estonia) using a procedure that was shown in detail in a previous study [11]. To remove excess EMIMCl, the MC-MCNT fibers were washed with ethanol several times and then dried in an oven at 40 °C at 2 mbar. The MC-MCNT fiber had a cylindrical form with a diameter of 965 ± 75 µm and a length of 4.5 ± 0.3 mm. The diameter of the MC-MCNT fibers was measured with a screw gauge (Eisco Labs, Rochester, NY, USA) after they had been stored in different electrolytes for 24 h. The applied electrolytes (0.1 M) were dissolved in propylene carbonate (PC, 99%) with the chosen salts, 1-ethyl-2,3-dimethylimidazolium trifluoro-methanesulfonate (EDMICF_3_SO_3_, EDMITF, 95%), lithium triflouro-methanesulfonate (LiCF_3_SO_3_, LiTF, 99%), and tetrabutylammonium triflouro-methanesulfonate (TBACF_3_SO_3_, TBATF, 98%). The MC-MCNT fibers applied in this research weighed 1.2 ± 0.1 mg with an estimated MCNT weight of 500 ± 48 µg, resulting in a density of MC-MCNT fibers in the range of 0.33 ± 0.03 g cm^−3^. To ensure reproducibility, at least three fibers were formed independently of each other and investigated. The EMIMCl, PC, EDMITF, LiTF, TBATF, and ethanol (technical degree) were obtained from Sigma-Aldrich (Taufkirchen, Germany) and used as supplied.

### 2.2. Linear Actuation Measurements

An internally developed linear muscle analyzer, coupled with custom software [20], was utilized to explore electromechanical deformation (EMD) measurements. This analytical tool comprises a movable stage integrated with a TRI202PAD force sensor (Panlab, Barcelona, Spain), onto which MC-MCNT samples were affixed. An arm featuring gold contacts, which serves as the working electrode, is positioned opposite this stage. These measurements occurred within a three-electrode cell containing electrolytes, with a platinum counter electrode and an Ag/AgCl (3M KCl) reference electrode. The signals from this cell’s three electrodes are interfaced with a Biologic PG581 potentiostat (Seyssinet-Pariset, France). Real-time measurements occur through a combination of the linear muscle analyzer and proprietary software designed for these specific measurements [20]. The change of mass (calculated to stress σ = mass change × 9.81 (gravimetrical constant)/(cross-section area π × r^2^ (r radius of fiber)) was conducted with a constant length of fiber between clamps of 1 mm, and the change of length (strain ε (%) =Δl/l × 100) was obtained with a constant force of 0.5 mN applied on the fixed fiber sample. To assure that no irreversible swelling of the MC-MCNT fiber occurred during measurements, the samples were fixed in the linear muscle analyzer and stretched in the range of 0.2% in the electrolytes (EDMITF, LiTF, and TBATF) in propylene carbonate 24 h prior to measurements. Different electrochemical techniques, such as cyclic voltammetry (scan rate 5 mV s^−1^) and square wave potential steps at frequencies 2.5 mHz to 100 mHz were conducted in a potential range of 0.7 V to −0.2 V for each applied electrolyte. Long-term measurements were performed at 100 mHz for 200 cycles, showing stress results. From the integration of the current density–time curves at each frequency, the diffusion coefficient *D* at positive potential (pos pot) and negative potential (neg pot) were obtained from Equations (1) and (2) [21].

(1) $$ln\left(1-\frac{Q}{Q_{t}}\right)=-b\cdot t$$

(2) $$D=\frac{b\cdot h^{2}}{2}$$

The expression *ln* (1 *− Q*/*Q_t_*) with *Q* is the charge at each time divided through the total charge *Q_t_* on the left side of Equation (1) plotted against time *t* at each applied frequency with the slope *b* obtained from previous research [22]. With the thickness *h* (cross-section of fiber) and slope *b*, the diffusion coefficients at positive potential D_pos.pot._ and negative potential D_neg.pot._ are obtained.

Chronopotentiometric (square wave current) measurements of MC-MCNT fibers were performed at applied current densities ±0.025 A g^−1^, ±0.05 A g^−1^, ±0.1 A g^−1^, ±0.25 A g^−1^, ±0.5 A g^−1^, and ±1 A g^−1^ with the same charge density of ±5 C g^−1^.

### 2.3. Characterizations

The surface and cross-section images of MC-MCNT fibers were taken by SEM (Tescan Orsay Holding, Brno-Kohoutovice, Czech Republic). The element content of MC-MCNT fiber after linear actuation cycles (different electrolytes) was analyzed using EDX spectroscopy (EDX, Oxford Instruments with X-Max 50 mm^2^ detector, High Wycombe, PA, USA) applying a cross-section image with polarization at the potentials 0.7 V and −0.2 V for 3 min. FTIR measurements (4000–500 cm^−1^) were performed using a Bruker Alpha with Platinum ATR spectrometer (Bruker Alpha, Billerica, MA, USA), and Raman spectroscopy (1800–1200 cm^−1^) was performed by applying an argon-ion laser (514 nm) from a Renishaw inVia micro-Raman spectrometer (Renishaw plc, resolution 2 cm^−1^, Wotton-under-Edge, UK). The resistivity of fiber samples was obtained using a digital multimeter (LCR200 Meter, EXTECH instruments, Nashua, NH, USA) with the determination of electronic conductivity σ_e_ (Equation (3) [23]), with l being the length of the fiber and A the cylindrical surface area (2∙π∙r∙*l* + 2∙π∙r^2^ with radius r and length *l* of fiber).

(3) $$\sigma_{e}=\frac{l}{R\cdot A}$$

## 3. Results and Discussion

Previous research [11] discovered that the potential range is qualitative in viewing which actuation direction is preferred. The actuation direction, either expansion at positive charging or negative charging, considering which ions lead to forming the electrical double layer, has been shown in MCNT yarns, also depending on the size of applied ions [24]. At first, we want to compare the linear actuation properties by applying cyclic voltammetry and square wave potential steps to analyze whether different applied electrolytes (cations) in their actuation responses can be differentiated. Chronopotentiometric measurements are conducted to analyze their sensor properties regarding different cations. The characterization of MC-MCNT is performed by using FTIR and Raman spectroscopy. BET (Braunauer–Emmett–Teller) measurements of MC-MCNT fiber have been made in recent research [25], revealing a BET-specific surface area of 101.62 m^2^ g^−1^, comparable to previous research [26] where 70 wt.% MCNTs were used. The average pore size distribution of MC-MCNT was 7.6 nm [25].

### 3.1. MC-MCNT Fiber Formulation and Characterization

**Figure 1:**
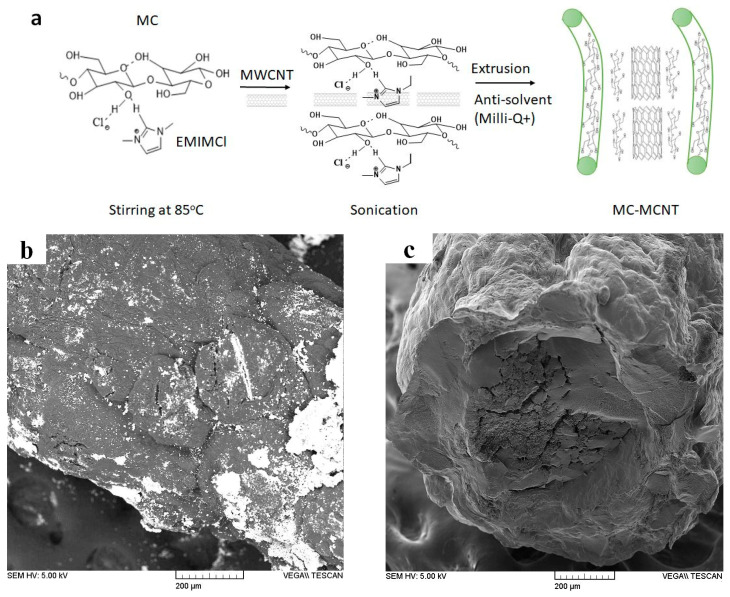
A schematic view of MC-MCNT formation is shown in (**a**), and SEM images (scale 500 µm) of MC-MCNT fiber are presented in (**b**). The SEM cross-section image (scale bar 200 µm) of MC-MCNT is presented in (**c**).

Figure 1a shows the scheme of MC-MCNT fiber production with MC-MCNT fiber SEM images (Figure 1b), and a cross-section image is presented in Figure 1c.

In general, microcrystalline cellulose (MC) is dissolved in Ionic liquids (EMIMCl) [27], as shown in Figure 1a, where the intense ions of the EMIMCl, especially their anions, break the hydrogen bonds [28]. In the second step, the MCNTs are included (50 wt.%), the suspension is sonicated, and the solution is directly placed in a syringe. To restore the hydrogen bonds, the MC-MCNT suspension was extruded, and fiber was formed in an anti-solvent (Milli-Q+ water) via the procedure shown in previous research [14]. The SEM fiber image in Figure 1b and the cross-section image shown in Figure 1c represent the MC-MCNT fiber. Based on previous research [29], we applied 10 wt.%, which led to more homogenously distributed MCNTs in the fiber, while the 50 wt.%, showing a more compact structure with MCNTs, was mainly found in the center of the MC-MCNT fiber [10] (Figure 1c). The main reason for having a more compact section in the middle of the MC-MCNT fiber was shown in previous research [30]. With higher MCNT loading up to 20 wt.%, the MCNTs tend to bundle in the inner core. The electronic conductivity of MC-MCNT fiber was found in the range of 1.25 ± 0.1 mS cm^−1^, similar to those studied before [11]. An electronic conductivity of 0.8 mS cm^−1^ was shown in former research [31] if cellulose with MCNTs (4 wt.%) was formed via electrospinning. We tried to electrospin our MC-MCNT but were unable to achieve any results. Increasing the hydrogen bond moieties of cellulose via modification revealed that, if loaded with 30 wt.% MCNTs, there is an electronic conductivity of up to 10 S cm^−1^ [32].

**Figure 2:**
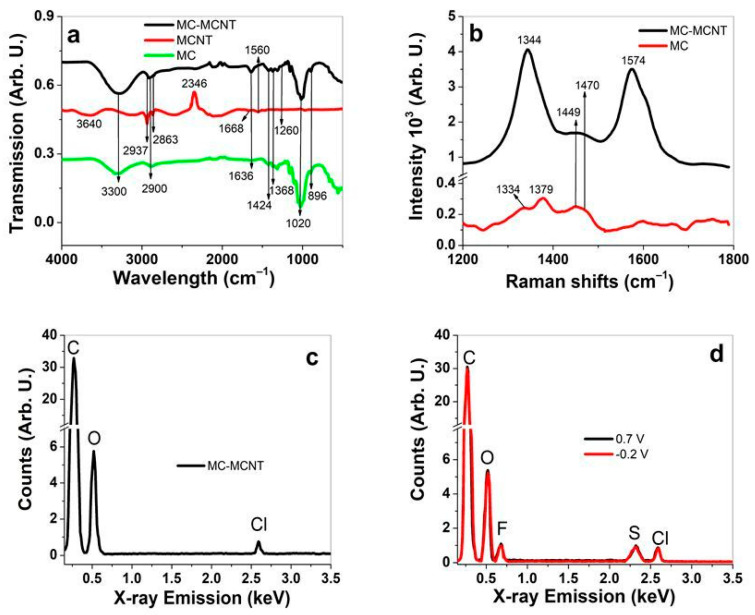
FTIR measurements (4000–500 cm^−1^) of MC-MCNT (black line), MCNT (red line), and MC (green line) are shown in (**a**). Raman spectroscopy (514 nm, 1800–1200 cm^−1^) of MC-MCNT (black line) and MC (red line) is displayed in (**b**). EDX spectroscopy (cross-section image of Figure 1c) directly after fiber assembly is presented in (**c**), and after the charging (0.7 V, black line) and discharging (−0.2 V, red line) of MC-MCNT in EDMITF–PC is shown in (**d**).

Further characterization is performed with FTIR measurements of MC-MCNT, MCNT, and MC, with results shown in Figure 2a. Additionally, Raman spectroscopy of MC and MC-MCNT is presented in Figure 2b. EDX measurements were taken from the cross-section image inner section in Figure 2c to determine the element composition of MC-MCNT fiber directly after formation. The EDX spectra after the charging (0.7 V) and discharging (−0.2 V) of EDMITF in PC are displayed in Figure 2d. The MC-MCNT fiber applying electrolytes LiTF and TBATF after linear actuation looks very similar in their EDX spectra, and the results are shown in Figure S1a and Figure S1b, respectively.

Typical FTIR bands (Figure 2a) of MC and MC-MCNT are shown at 3300 cm^−1^, which refers to the stretching vibrations of OH– bonds [33], with the same bands shifted to 3630 cm^−1^ shown in MCNTs. The shifting to a lower wavenumber for MC-MCNT proves stronger hydrogen bondings [34]. The typical C–H stretching vibration bands for MCNTs are shown at 2937 cm^−1^ and 2863 cm^−1^ and are also found in MC-MCNT, revealing that MCNTs are incorporated in MC. The adsorption band at 2346 cm^−1^ is the backbone of the MCNTs. As was shown in previous research [35], with the functionalization of MCNTs, this band decreases in intensity. Additional MCNT bands are shown at 1668 cm^−1^ (skeletal motions of MCNTs [36]), 1560 cm^−1^ (C=C stretching modes [36]), and 1260 cm^−1^ (C–O stretching mode), with both bands also reflected in MC-MCNT. MC bands [37,38] in MC-MCNT are found at 2900 cm^−1^ (C–H stretching vibration), 1636 cm^−1^ (vibration of water molecules absorbed in MC), 1424 cm^−1^ (bending vibration of CH_2_), 1368 cm^−1^ (O–C=O stretching vibration [34]), and 1020 cm^−1^ (stretching vibrations of C–O bonds), and the 896 cm^−1^ belongs to C–H vibration.

The Raman spectroscopy (Figure 2b) reveals the typical strong C–C in plane bands [39] with the D peak at 1344 cm^−1^ and the G peak at 1574 cm^−1^. MC bands [40] are fragile, reaching 1369 cm^−1^ (MC backbone) and 1334 cm^−1^ (C–H bending vibration). The 1449 cm^−1^ (HCH bending vibration [41]) and 1470 cm^−1^ (CH_2_ bending vibration) signals of MC are somehow covered by the broadband (between 1436 and 1471 cm^−1^) of MC-MCNT. The FTIR and Raman spectroscopy show that MCNTs are incorporated in MC-MCNT fiber.

**Figure 3:**
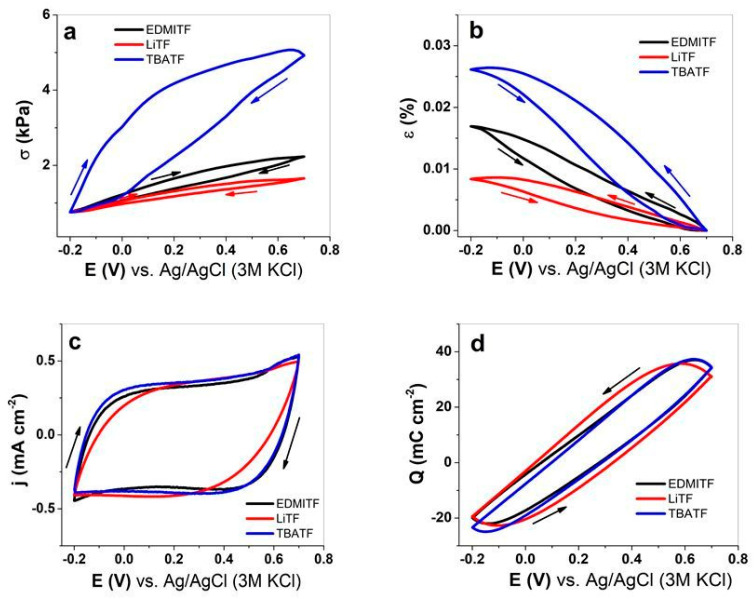
Cyclic voltammetry (scan rate 5 mV s^−1^, 2nd–3rd cycle) of MC-MCNT fiber using three different electrolytes in PC solvent, EDMITF (black line), LiTF (red line), and TBATF (blue line). The stress σ against potential range E (0.7 V to −0.2 V) is shown in (**a**), the strain ε in (**b**), the current density j in (**c**), and the charge density Q in (**d**). The arrows indicate the direction of the scan.

Further analysis is conducted via EDX spectroscopy to see the elemental composition (directly after the regeneration and cleaning procedure of MC-MCNT) by taking the inner center of the fiber, with spectra shown in Figure 2c. Typical element carbon (C) signals are shown at 0.27 keV, oxygen at 0.52 keV, and chloride at 2.59 keV. The chloride peak refers to the EMIMCl that is applied to dissolve MC, the residue left after washing. To demonstrate which elements are still present at the charging and discharging process, Figure 3d shows the results of MC-MCNT in EDMITF–PC electrolyte. Additional fluoride (F) elements are shown at 0.68 keV and sulfur (S) at 2.32 keV, which do not alter at charging/discharging. The EDX spectra of LiTF (Figure S1a) and TBATF (Figure S1b) show a similar tendency. Former research [42] investigated MWCNT fiber using TBATF–PC electrolyte, revealing that expansion occurs during discharging. It explained that the TF^−^ anions incorporated in the MCNT network and cations (TBA^+^) form the EDL. Recent research [25] using LiTFSI (bis(trifluoromethane)sulfonimide lithium salt) electrolyte in PC solvent showed a similar tendency of MC-MCNT element detection, with no alteration of sulfur, chloride, and fluoride found at charging/discharging. Therefore, we assume that the TF^−^ is incorporated in the MCNTs of MC-MCNT fiber and affects the linear actuation direction, with further investigation shown in the next section.

### 3.2. Linear Actuation of MC-MCNT

The structure of MC-MCNT with MCNTs located in the center of the fiber follows the electrical double-layer (EDL) mechanism over charge injection (non-faradaic process [12]), causing the C–C bond length changes [13]. In this work, the applied electrolytes with the same anions (TF^−^) and those shown by EDX spectroscopy (fluoride) tend to stay immobile in the MC-MCNT fiber. Therefore, the applied cations forming the EDL and the extent of linear actuation in stress and strain are investigated. To ensure the reproducibility of the linear actuation behavior of MC-MCNT fiber, at least three for each applied electrolyte are investigated, with results in mean values, including standard deviations.

#### 3.2.1. Cyclic Voltammetry

The linear electromechanical deformation (EMD) measurements of MC-MCNT fibers are performed in EDMITF, LiTF, and TBATF electrolytes using PC as the solvent. The cyclic voltammetry (scan rate 5 mV s^−1^) of stress against potential E is shown in Figure 3a, strain in Figure 3b, current density j (CV shapes) in Figure 3c, and the coulovoltammetric response (charge density potential curves) is presented in Figure 3d.

The stress shown in Figure 3a is always opposite to strain (Figure 3b); contraction in stress means expansion in strain. Comparing the three electrolytes, all have their main expansion at discharging, following the order TBATF > EDMITF > LiTF. Former research [24] studying MWCNT yarn in tetrabutylammonium hexafluorophosphate (TBAPF_6_) in acetonitrile showed the main expansion at discharging. The main explanation was given that either anions or cations are influenced by van der Waals volume and the porosity of the electrodes as well as applied ion concentration [43]. Therefore, the ions’ size in the applied different cations is crucial in influencing linear stress and strain responses. Our research has the same anions, TF^−^, with a van der Waals volume of 80 Å^3^. The solvent PC also plays a role, especially due to its high viscosity of 2.513 Pa s; the mobility of TF^−^ anions is reduced, as they also have a weak solvation shell [44]. The specialties of TF^−^ anions are the size of the anion, the non-spherical form in a cylindrical shape, and the delocalized charge [45], which is trapped more easily in nano-pores than other anions. Table 1 compares the stress and strain numbers, the cations’ van der Waals volume (V.D.W.V.), and the solvation numbers in PC of the electrolytes EDMITF, LiTF, and TBATF.

The solvation number of EDMI^+^ is not reported in the literature, but recent research [47] revealed a weak rather than strong solvation in PC. In the case of Li^+^ ions, former research showed that, by using SWCNT sheets and applying acetonitrile as a solvent, the ions moved in without solvation shells [43]. Therefore, we can concentrate on cation size to compare the linear actuation of MC-MCNT, which was found to be the largest in TBATF, with nearly 1.8 times higher stress than EDMITF (cation size TBA^+^ 2.2 times larger than EDMI^+^). The LiTF showed the lowest stress of MC-MCNT fiber, which is also explainable by Li^+^ ions having the smallest van der Waals volume (Table 1). The current density potential curves (Figure 3c) show capacitive shapes for all applied electrolytes. The coulovoltammetric response (charge density potential curve) in Figure 3d has a close loop for all applied electrolytes in the potential range 0.7 V to −0.2 V, meaning that charging/discharging is in balance. The charge densities have only minor differences, with TBATF having 58.8 ± 4.3 mC cm^−2^, followed by EDMITF with 56.1 ± 4.1 mC cm^−2^, and the lowest being found for LiTF with 50.2 ± 3.6 mC cm^−2^. As shown in previous research [11,14,25] using Cell-CNT fiber, the applied electrolytes, the potential ranges, and the solvent play a role in the actuation direction (cation or anion forming the electrical double layer). When comparing this work’s potential range of 0.7 V to −0.2 V to the former’s [11] potential range of 0.8 V to −0.3 V, the discharging expansion was found to be comparable if the same cation, Li^+^, was used. The other electrolytes that differ in size surely have an increased linear actuation in strain and stress.

#### 3.2.2. Square Wave Potential Steps of MC-MCNT Fiber

**Figure 4:**
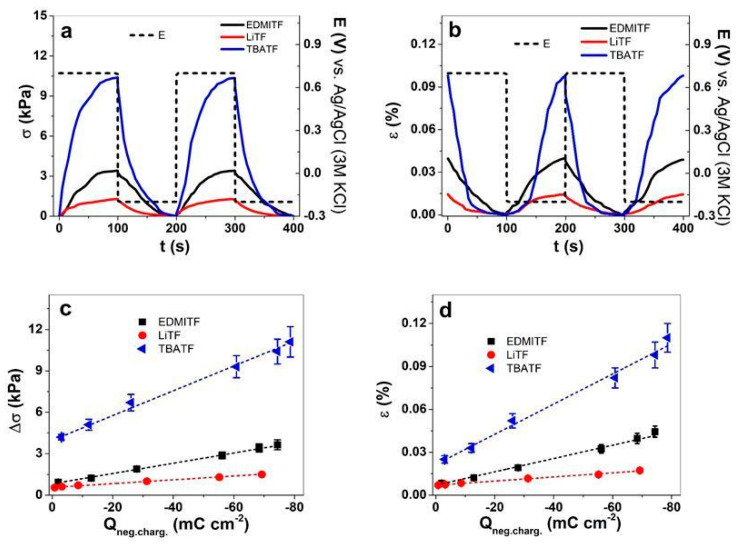
Square wave potential steps of MC-MCNT fiber applying three different electrolytes in PC solvent, EDMITF (black line, ■), LiTF (red line, ●), and TBATF (blue line, ◄), showing two subsequent cycles (3rd–4th) at 5 mHz at potential range E (dashed black line) of 0.7 V to −0.2 V of stress σ (lowest values set to zero) in (**a**) and strain ε against time t in (**b**). The stress difference Δσ against charge density at negative charging (Q_neg.charg._) is presented in (**c**) and the strain ε in (**d**). The dashed lines in (**c**,**d**) represent the linear fit.

A different electrochemical technique combined with linear measurements is applied, called chronoamperometric measurements (square wave potential steps). MC-MCNT fiber in electrolytes EDMITF, LiTF, and TBATF is investigated at applied frequencies of 2.5 mHz to 100 mHz. The stress and strain curves at 5 mHz are presented in Figure 4a and Figure 4b, respectively. The current density–time curves (Figure S2a) at each applied frequency were calculated; the stress against charge density at negative charging Q_neg.charg._ of MC-MCNT is shown in Figure 4c, and the strain is presented in Figure 4d. For each applied electrolyte, at least three different measurements from each formulated MC-MCNT fiber are used, with results presented in mean values with standard deviations. The stress differences against applied frequencies are shown in Figure S2b, and the strain is presented in Figure S2c.

The stress and strain time curves (5 mHz) shown in Figure 4a,b reveal a similar tendency to cyclic voltammetry (Figure 3a,b). MC-MCNT fiber with the electrolyte TBATF showed the best stress of 10.4 kPa and strain of 0.098%. EDMITF had 3 times lower stress with 3.4 kPa stress and 2.2 times reduced strain of 0.044%. LiTF electrolytes showed the lowest values, with 1.3 kPa and 0.014% strain. The stress difference Δσ (Figure S2b) and strain ε (Figure S2c) revealed a general trend; the stress and strain decreased with increasing frequencies. Due to the shorter time, there is less TF^−^ ingress at an increasing frequency, with the formation of the EDL over ion injection [48] being carried out by the cations on the interface of bundled MC-MCNT (we assume on the inner core of MC-MCNT, shown in Figure 1c). For a longer time (low frequencies), more TF^−^ goes into deeper pores of MC-MCNT fibers with cations forming the EDL, leading to a more significant linear actuation response, with a similar tendency shown in the MWCNT actuator [49]. The stress differences and strain against negative charging Q_neg.charg._ in Figure 4c,d reveal a linear functionality for MC-MCNT fiber in the three different electrolytes. At frequency 2.5 mHz (charge density −78.6 mC cm^−2^), the stress of MC-MCNT in TBATF showed 11.1 ± 1.1 kPa, with strain reaching the best values of 0.11 ± 0.01%. In the case of EDMITF, the charge density was −74.4 mC cm^−2^ with stress values at 3.65 ± 0.32 kPa and strain at 0.044 ± 0.004%. MC-MCNT fiber had reduced stress in LiTF at a charge density of −69.2 mC cm^−2^, showing stress of 1.5 kPa and strain of 0.017%. The charge density at negative polarization found for MC-MCNT fiber in the TBATF electrolyte was 1.05 times higher compared to EDMITF and 1.13 times higher if compared with LiTF. The slightly higher charge density of MC-MCNT fiber in TBATF cannot fully explain the much higher strain and stress. Figure S3a shows the diffusion coefficients at a negative potential (−0.2 V) D_neg.pot._, and Figure 3b shows the diffusion coefficients at positive potential D_pos.pot._ of MC-MCNT fiber in the three different electrolytes, obtained from Equations (1) and (2). The general tendency shows that the diffusion coefficients also increased with increasing frequency. The reason for this relies on the time provided being shorter (as an example, at 0.1 Hz) for ion injection and, therefore, reduced charge injection, leading to an increased diffusion coefficient of ions [29]. In the case of a longer time (low frequency, 2.5 mHz), deeper ion injection and other processes in MC-MCNT fiber occur, such as relaxations [50], leading to higher charge injection that influences ion diffusion and linear actuation response. In the case of TBATF, the MC-MCNT showed higher linear actuation than if EDMITF or LiTF was applied. The diffusion coefficients (Figure S3a) of TBATF at negative potential D_neg.pot._ showed at 2.5 mHz 0.789 ± 0.07 10^−7^ cm^2^ s^−1^ with a 1.16 times lower value for EDMITF (0.675 ± 0.06 10^−7^ cm^2^ s^−1^) and 2 times reduced diffusion coefficient for LiTF (0.389 ± 0.04 10^−7^ cm^2^ s^−1^). A similar tendency is shown for the diffusion coefficients at positive potential D_pos.pot._ in Figure S3b. In addition to the cation sizes, their hydrophilic/hydrophobic properties must be considered, with EDMI^+^ being weak hydrophobic [51], TBA^+^ being strongly hydrophobic [52], and Li^+^ ions tending to be more hydrophilic. Li^+^ ions have a solvation shell in PC, but, if they enter the MC-MCNT fiber, forming an EDL on the inner core, the solvation shell is not transported within [43]. TBA^+^ and EDMI^+^ cations move without a solvation shell in PC, and, if they enter MC-MCNT fiber, the outer surface that contains more MC is hydrophilic, while the inner core, where the most concentrated MCNTs are, is more hydrophobic. Therefore, TBA^+^ and EDMI^+^ are assumed to move slightly faster in MC-MCNT than Li^+^, as shown by the tendency in Figure S3a and Figure S3b, respectively.

**Figure 5:**
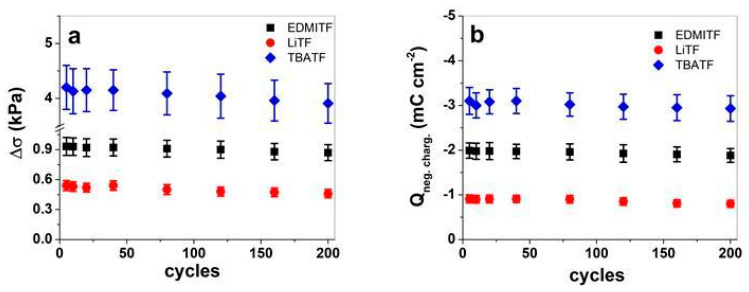
Square wave potential step in long-term measurements at 0.1 Hz of MC-MCNT fiber using three different electrolytes in PC solvent, EDMITF (■), LiTF (●), and TBATF (◄), in potential range 0.7 V to −0.2 V. The stress difference Δσ against cycles is shown in (**a**), and the charge density at negative charging Q_neg.charg._ against cycles is shown in (**b**).

Further studies regarding long-term measurements (200 cycles, 0.1 Hz) of MC-MCNT fibers are conducted to investigate which ions show the best stability because of the best stress. The stress–time curves are presented in Figure S4a for EDMITF, Figure S4b for LiTF, and Figure S4c for TBATF. The stress differences against cycle numbers are shown in Figure 5a, and the charge density Q_neg.charg._ is shown in Figure 5b.

The stress–time curves of MC-MCNT fiber shown in Figure S4a–c have differences in amplitude, but a slight creep (shifting of baseline) was detected for EDMITF at the range of 4.6% (Figure S4a), and LiTF (Figure S4b) had 9% creep. The electrolyte TBATF showed the best stress in MC-MCNT fiber and, after 200 cycles, had no creep development (Figure S4c). Recent research investigating MCNT fibers showed creep development with the explanation given that the MCNTs in MC-MCNT fibers experience a sliding effect under load [53]. The main reason why LiTF has much higher creep in MC-MCNT fiber is not fully understood and needs more investigation. The stress difference Δσ shown in Figure 5a had the best values for MC-MCNT fiber in TBATF with 4.20 ± 0.40 kPa (cycle 5) and decreased at cycle 200 to 3.91 ± 0.36 kPa (decrease in the range of 6.9%). The EDMITF electrolyte in MC-MCNT fiber had 0.93 ± 0.09 kPa (cycle 5) with a decrease of 6.7% at cycle 200. The electrolyte LiTF showed the lowest stress in MC-MCNT fiber of 0.54 ± 0.05 kPa with a 14.8% decrease at cycle 200. The charge density at long-term cycling (Figure 5b) with the highest charge for MC-MCNT fiber was found in TBATF electrolyte (−3.1 ± 0.3 mC cm^−2^), followed by −2.0 ± 0.2 mC cm^−2^ for EDMITF, and the lowest was found for LiTF in the range of −0.91 ± 0.08 mC cm^−2^. The general trend of linear actuation of MC-MCNT revealed that the charge injection determines the linear actuation properties.

In summary, from linear actuation measurements, the MC-MCNT fibers mainly expand at the negative potential for all applied electrolytes. The cations of the electrolyte, in order TBA^+^ > EDMI^+^ > Li^+^, follow from high to low in stress, strain, charge density, cycle stability, diffusion coefficients, ion size, and hydrophobicity. If it is possible to identify the cations from the linear actuation studies, an actuator–sensor device can be obtained. The sensor functionality is investigated with chronopotentiometric studies.

### 3.3. Sensor Properties over Chronopotentiometric Measurements

If we consider Le Chatelier’s principle that if a system is in electrochemical balance, often applied for faradaic actuators [54], additional parameters either in current or potential evolution can be measured simultaneously to their actuation response, shown as well in previous research [55], on SWCNT, being adaptable in dual sensing and actuation. MC-MCNT fiber has also been shown in recent research [14] to have anions and cations that can be selectively detected by switching the potential range in aqueous electrolytes. Cellulose MCNT-forming fiber over electrospinning showed a sensor for different ions and ion concentrations in aqueous electrolytes [56]. Other research showed that MCNTs with cellulose could functionalize as a water sensor [57] or chemical vapor such as methanol and 1-butanol [58]. Not much research has been carried out for cation detection regarding their size in organic solvents such as PC using MC-MCNT fiber.

The MC-MCNT fiber in three different electrolytes (EDMITF, LiTF, TBATF) has the same charge density of ±5 C g^−1^ with current densities ±0.025 A g^−1^, ±0.05 A g^−1^, ±0.1 A g^−1^, ±0.25 A g^−1^, ±0.5 A g^−1^, and ±1 A g^−1^. Our main goal is to investigate whether the cations from electrolytes can be differentiated from each other. The obtained potential time curves at each applied current density (*i*/*m*, *i* is current and *m* is the mass of MCNTs in MC-MCNT fibers) with the integration of the discharged curve (*E* as potential against time *t*) and the electrical energy *U_e_* as another sensing parameter can be calculated (Equation (4)).

(4) $$U_{e}\left(t\right)=\frac{i}{m}\int E\left(t\right)dt$$

**Figure 6:**
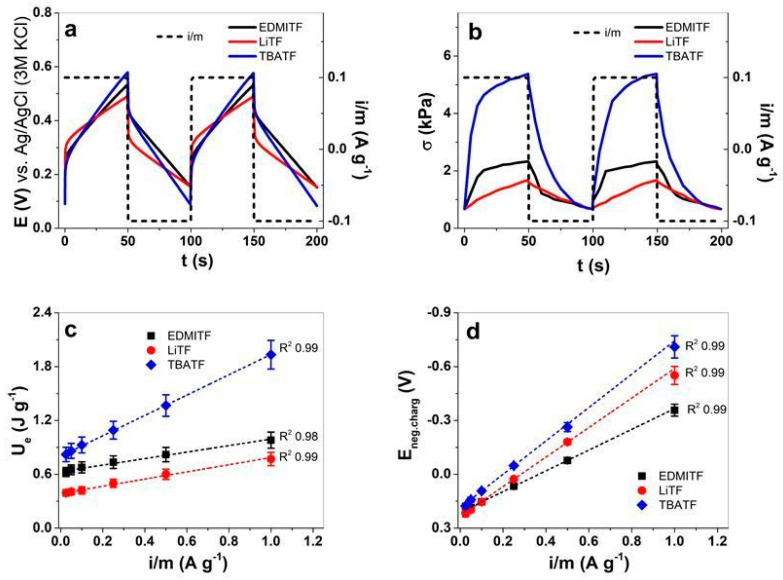
Chronopotentiometric measurements (current densities of ±0.025–±1 A g^−1^, constant charge of ±5 C g^−1^) of MC-MCNT fiber in three different electrolytes, EDMITF (black line, ■), LiTF (red line, ●), and TBATF (◄), using PC as solvent showing the potential time curves (two subsequent cycles, 4th to 5th) at ± 0.01 A g^−1^ (dashed black line) in (**a**) and the stress curves in (**b**). From Equation (4), the electrical energy U_e_ against current density i/m is shown in (**c**), and the maximum potential at negative charging E_neg.charg._ is presented in (**d**). The dashed lines in (**c**,**d**) represent the linear fit with a correlation coefficient R^2^ of 0.98–0.99.

The potential time curves at applied current density ±0.1 A g^−1^ of the MC-MCNT fiber in the three different electrolytes are shown in Figure 6a. The linear actuation in stress at the same current density is presented in Figure 6b, and the stress variation at each applied current density is shown in Figure S5a. The electrical energy *U_e_* against the current density *i*/*m* is presented in Figure 6c, the potential evolution at negative charging E_neg.charg._ is shown in Figure 6d, and those at positive charging E_pos.charg._ are displayed in Figure S5b.

The potential time curves shown in Figure 6a reveal each electrolyte in PC applied in MC-MCNT fiber in different shapes and forms regarding potential evolution at charging/discharging. For each applied electrolyte, the two subsequent cycles in Figure 6a are concurrent, revealing that charging/discharging is in balance, which is required to obtain reliable sensors. Figure 6b shows, at the same applied current density, the linear actuation properties in stress against time, revealing a similar observation as shown before: that MC-MCNT fiber has the best actuation with its main extension at discharging in the following order of electrolytes in PC: TBATF (4.65 kPa) > EDMITF (1.67 kPa) > LiTF (0.98 kPa). With the same wire in general to measure the linear actuation of MC-MCNT fiber, additional parameters such as current and Equation (4), the electrical energy U_e_ can be calculated to differentiate the cations of the applied electrolytes. Either electrical energy (Figure 6c), potential evolution at negative charging E_neg.charg._ (Figure 6d), stress response (Figure S5a), or potential evolution at positive charging E_pos.charg._ (Figure S5b) against current density i/m is applied. To obtain the sensor sensibility, the linear fit equations (y = a + b·x, with “b” as the slope, the intercept as “a” and “x”, and the current density *i*/*m* = *j*) of the MC-MCNT fiber for each applied electrolyte are shown in Table 2.

The linear fit equations shown in Table 2 reveal that the cations TBA^+^ (TBATF) and Li^+^ (LiTF) of MC-MCNT fiber can be differentiated for each shown equation. In the case of EDMI^+^, which is weakly hydrophobic, and Li^+^ (hydrophilic), only the electrical energy U_e_, E_neg.charg._, and the stress show some differences and can be differentiated from each other. Regarding this investigation, the cations from the three different electrolytes can be sensed, forming a cation-selective electrode in an organic solvent. Previous research [50] showed that SWCNT-PVdF(HFP) films can sense temperature and electrolyte concentration, mostly shown in aqueous solutions. The authors draw these sensing properties to faradaic components in the non-faradaic process of CNT materials. Other work using cellulose–MCNT composites showed multifunctional sensor ability for temperature, tensile strain, humidity, and liquid water [31]. The use of MC-MCNT fiber as an ion (cation) detector in organic electrolytes has not been shown; mainly, using such composites as a vapor sensor has been investigated [59]. Hazardous material detection, such as mercury detection in acetonitrile using an MCNT Ionic liquid paste electrode, did reveal sensor selectivity [60]. The detection of ions in organic solvents of MC-MCNT fiber will enable applications of smart materials as it is an actuator or sensor for potentiometric detection of ions [19] in non-aqueous solvents, with ion-selective electrodes being especially interesting for applications in industry for contamination of organic solvents.

## 4. Conclusions

MC-MCNT fiber with MCNTs (50 wt.%) made via extrusion is applied in this research using three different electrolytes, EDMITF, LiTF, and TBATF, in a potential range of 0.7 V to 0.2 V for linear actuator and sensor response in PC solvent. SEM images with MCNT characterize the MC-MCNT fiber found mainly in the center of the fiber. FTIR and Raman spectroscopy showed characteristic signals of microcrystalline cellulose (MC) and MCNTs, which were found as well in the MC-MCNT composite. Each electrolyte showed its main expansion at discharging, with the TF^−^ ions incorporated in MC-MCNT fiber (EDX analysis), leading to mainly cation influence forming the EDL at discharging. The cations of the electrolytes, in terms of their size, stress and strain, diffusion coefficients, charge density, and long-term measurements, are found to be in the following order: TBA^+^ (TBATF) > EDMI^+^ (EDMITF) > Li^+^ (LiTF). With cation-selective electrodes (MC-MCNT fiber), the sensor ability is investigated via chronopotentiometric measurements, differentiating the cations (EDMI^+^, Li^+^, and TBA^+^) in PC. The electrical energy, the potential evolution at charging or discharging, or the linear actuator response can be applied to obtain sensor functionalities. Such dual actuator–sensor MC-MCNT devices can be used in actuators or as cation-selective electrodes in the potentiometric determination of ion contents in waste organic solvents, mainly for industrial purposes.

## Figures and Tables

**Table 1:** The comparison of stress, strain, and cation size (V.D.W.V.) and solvation number (n) of the three different applied electrolytes in linear actuation of MC-MCNT fiber.

Cations	MC-MCNT Linear Actuation		Cation Size + Solvation Number	
	Stress σ (kPa)	Strain ε (%)	V.D.W.V. (Å^3^) [46]	n
EDMI^+^	1.49 ± 0.12	0.0170 ± 0.001	133	n.A.
Li^+^	0.9 ± 0.07	0.0084 ± 5 × 10^−4^	1.84	3–4 [44]
TBA^+^	4.17 ± 0.31	0.026 ± 0.0022	293	0

**Table 2:** MC-MCNT fiber sensor equations of electrical energy U_e_ and potential at negative charging E_neg.charg._ and positive charging E_pos.charg._, as well as the linear actuation response regarding stress σ of the applied electrolytes EDMITF, LiTF, and TBATF.

Cations	*U_e_* (J g^−1^)	−E_neg.charg._ (V)	E_pos.charg._ (V)	σ (kPa)
EDMI^+^	0.63–0.36·*j* (A g^−1^)	−0.21–0.58·*j* (A g^−1^)	0.48–0.49·*j* (A g^−1^)	1.65 + 0.01·*j* (A g^−1^)
Li^+^	0.39–0.40·*j* (A g^−1^)	−0.23–0.82·*j* (A g^−1^)	0.41–0.71·*j* (A g^−1^)	1.02 + 0.03·*j* (A g^−1^)
TBA^+^	0.8–1.13·*j* (A g^−1^)	−0.19–0.93·*j* (A g^−1^)	0.52–0.66·*j* (A g^−1^)	4.72 + 0.01·*j* (A g^−1^)

## Data Availability

The data presented in this study are available on request from the corresponding author. The data are not publicly available due to ongoing research on this subject.

## Supplementary Materials

The following supporting information can be downloaded at: https://www.mdpi.com/article/10.3390/polym16030339/s1, Figure S1: EDX spectroscopy of MC-MCNT fiber at potential 0.7 V (black line) and −0.2 V (red line) presenting the electrolyte LiTF in (a) and TBATF in (b) with PC solvent; Figure S2: Square wave potential steps of MC-MCNT fiber in three different electrolytes using PC solvent such as EDMITF (black line, ■), LiTF (red line, ●) and TBATF (blue line, ◄) showing at 5 mHz frequency two subsequent cycles (3rd–4th) of current density j against time t with potential E (0.7 V to −0.2 V) in (a). The stress difference Δσ is shown in (b) and the strain ε in (c) against the applied frequencies f (0.0025 Hz to 0.1 Hz); Figure S3: Diffusion coefficient at negative potential D_neg.pot._ against applied frequencies f (0.0025 Hz to 0.1 Hz) of MC-MCNT fiber in three different electrolytes such as EDMITF (■), LiTF (●) and TBATF (◄) are shown in (a) and the diffusion coefficient at positive potential (D_pos.pot_.) is presented in (b). The dashed lines represent the linear fit; Figure S4: Stress time curves (200 cycles) at 0.1 Hz at a potential range of 0.7 V to −0.2 V of MC-MCNT fibers showing the electrolyte EDMITF in (a), LiTF in (b) and TBATF in (c) using PC as the solvent; Figure S5: Chronpotentiometric measurements of MC-MCNT fiber in EDMITF (■), LiTF (●), and TBATF (◄) electrolyte in PC solvent showing stress σ in (a) and positive charging potential E_pos.charg._ in (b) against current density *i*/*m* (±0.025 A g^−1^–±1 A g^−1^). The dashed lines represent the linear fit with a correlation coefficient R^2^ at 0.99.
