# Supplementary material for: Cation-Selective Actuator–Sensor Response of Microcrystalline Cellulose Multi-Walled Carbon Nanotubes of Different Electrolytes Using Propylene Carbonate Solvent
Source: Polymers (Basel). 2024 Jan 26;16(3):339. doi: 10.3390/polym16030339 (PMC10857484; doi:10.3390/polym16030339)
Supplement: Supplementary file 1 [file polymers-16-00339-s001.zip › polymers-2798424-supplementary.pdf]

Supplementary

Article

Cation-Selective Actuator–Sensor Response of Microcrystalline Cellulose Multi-Walled Carbon Nanotubes of Different Electrolytes Using Propylene Carbonate Solvent

Fred Elhi [1], Quoc Bao Le [2] and Rudolf Kiefer [2,*]

[1] Intelligent Materials and Systems Lab, Institute of Technology, University of Tartu, Nooruse 1, 50411 Tartu, Estonia; elhi.fred@gmail.com

[2] Conducting Polymers in Composites and Applications Research Group, Faculty of Applied Sciences, Ton Duc Thang University, Ho Chi Minh City 700000, Vietnam; lequocbao@tdtu.edu.vn

* Correspondence: rudolf.kiefer@tdtu.edu.vn; Tel.: +84-35-6301264

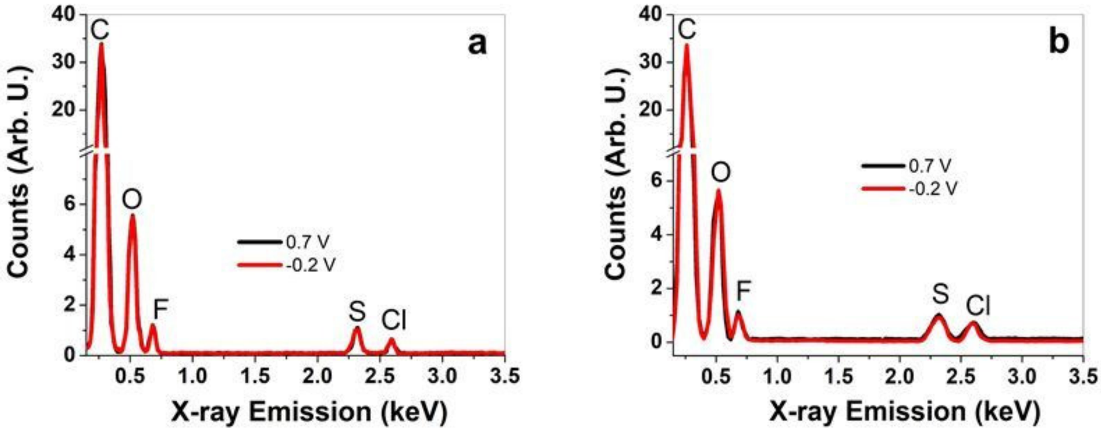

Figure S1. EDX spectroscopy of MC-MCNT fiber at potential 0.7 V (black line) and -0.2 V (red line) presenting the electrolyte LiTF in a) and TBATF in b) with PC solvent.

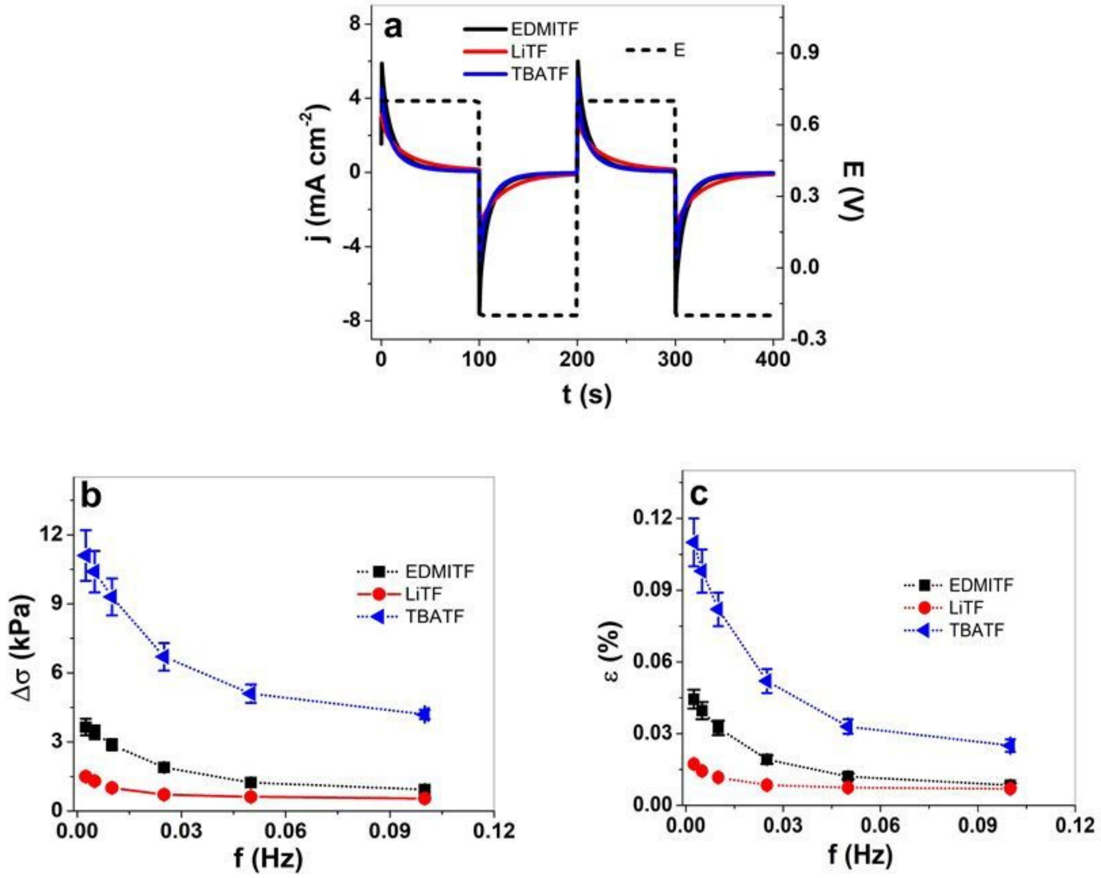

Figure S2. Square wave potential steps of MC-MCNT fiber in three different electrolytes using PC solvent such as EDMITF (black line, ■), LiTF (red line, ●) and TBATF (blue line, ◄) showing at 5 mHz frequency two subsequent cycles (3rd - 4th) of current density j against time t with potential E (0.7 V to -0.2 V) in a). The stress difference Δσ is shown in b) and the strain ε in c) against the applied frequencies f (0.0025 Hz to 0.1Hz).

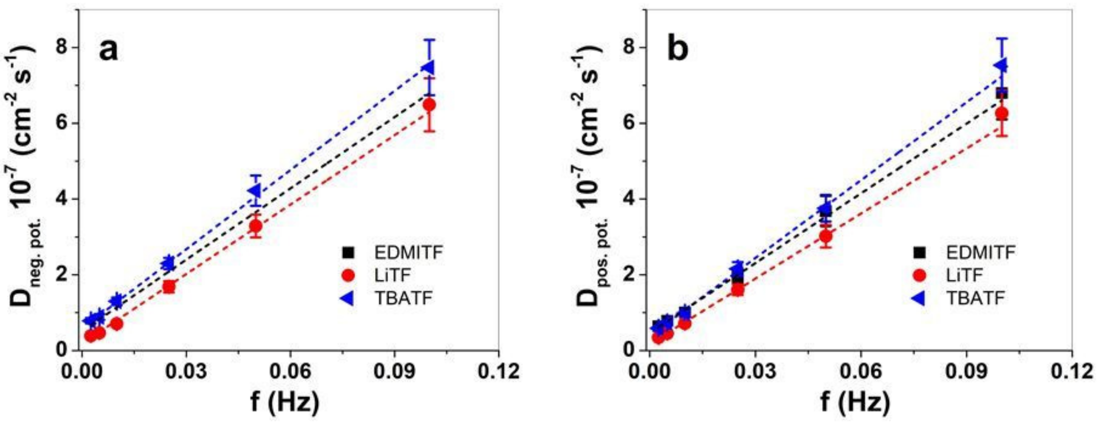

Figure S3. Diffusion coefficient at negative potential $D_{neg.\ pot.}$ against applied frequencies f (0.0025 Hz to 0.1 Hz) of MC-MCNT fiber in three different electrolytes such as EDMITF (■), LiTF (●) and TBATF (◀) are shown in a) and the diffusion coefficient at positive potential ($D_{pos.\ pot.}$) is presented in b). The dashed lines represent the linear fit.

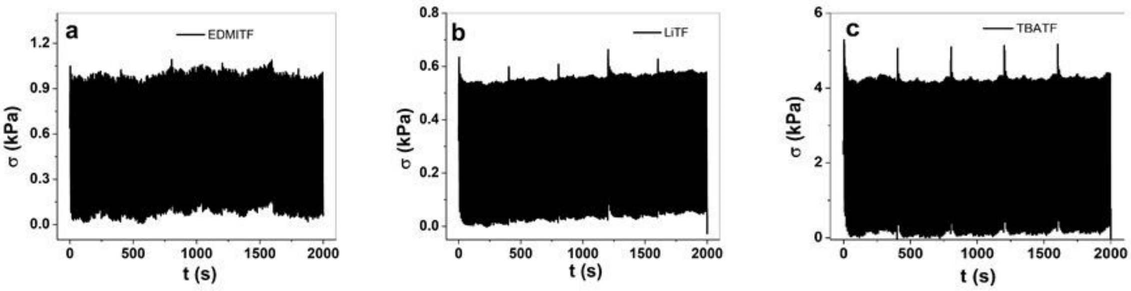

Figure S4. Stress time curves (200 cycles) at 0.1 Hz at a potential range of 0.7 V to -0.2 V of MC-MCNT fibers showing the electrolyte EDMITF in a), LiTF in b) and TBATF in c) using PC as the solvent.

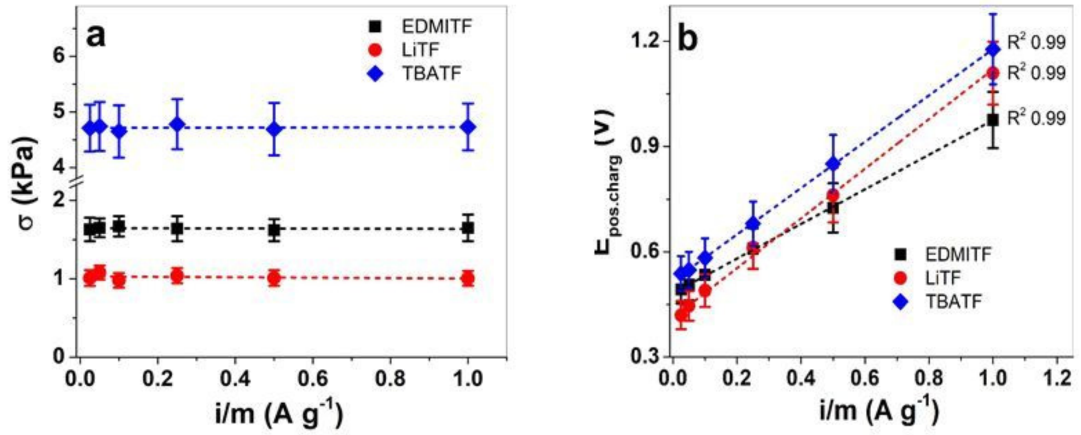

Figure S5. Chronpotentiometric measurements of MC-MCNT fiber in EDMITF (■), LiTF (●), and TBATF (◀) electrolyte in PC solvent showing stress σ in a) and positive charging potential $E_{pos\ charg}$ in b) against current density i/m (± 0.025 A g^{-1} - ± 1 A g^{-1}). The dashed lines represent the linear fit with a correlation coefficient R^2 at 0.99.
